# Four Weeks of IV Iron Supplementation Reduces Perceived Fatigue and Mood Disturbance in Distance Runners

**DOI:** 10.1371/journal.pone.0108042

**Published:** 2014-09-23

**Authors:** Amy Woods, Laura A. Garvican-Lewis, Philo U. Saunders, Greg Lovell, David Hughes, Ruth Fazakerley, Bev Anderson, Christopher J. Gore, Kevin G. Thompson

**Affiliations:** 1 University of Canberra, Research Institute for Sport and Exercise, Canberra, Australia; 2 Australian Institute of Sport, Canberra, Australia; West Virginia University School of Medicine, United States of America

## Abstract

**Purpose:**

To determine the effect of intravenous iron supplementation on performance, fatigue and overall mood in runners without clinical iron deficiency.

**Methods:**

Fourteen distance runners with serum ferritin 30–100 µg·L^−1^ were randomly assigned to receive three blinded injections of intravenous ferric-carboxymaltose (2 ml, 100 mg, IRON) or normal saline (PLACEBO) over four weeks (weeks 0, 2, 4). Athletes performed a 3,000 m time trial and 10×400 m monitored training session on consecutive days at week 0 and again following each injection. Hemoglobin mass (Hbmass) was assessed via carbon monoxide rebreathing at weeks 0 and 6. Fatigue and mood were determined bi-weekly until week 6 via Total Fatigue Score (TFS) and Total Mood Disturbance (TMD) using the Brief Fatigue Inventory and Brunel Mood Scale. Data were analyzed using magnitude-based inferences, based on the unequal variances t-statistic and Cohen's Effect sizes (ES).

**Results:**

Serum ferritin increased in IRON only (Week 0: 62.8±21.9, Week 4: 128.1±46.6 µg·L^−1^; p = 0.002) and remained elevated two weeks after the final injection (127.0±66.3 µg·L^−1^, p = 0.01), without significant changes in Hbmass. Supplementation had a moderate effect on TMD of IRON (ES -0.77) with scores at week 6 lower than PLACEBO (ES -1.58, p = 0.02). Similarly, at week 6, TFS was significantly improved in IRON vs. PLACEBO (ES –1.54, p = 0.05). There were no significant improvements in 3,000 m time in either group (Week 0 vs. Week 4; Iron: 625.6±55.5 s vs. 625.4±52.7 s; PLACEBO: 624.8±47.2 s vs. 639.1±59.7 s); but IRON reduced their average time for the 10×400 m training session at week 2 (Week 0: 78.0±6.6 s, Week 2: 77.2±6.3; ES–0.20, p = 0.004).

**Conclusion:**

During 6 weeks of training, intravenous iron supplementation improved perceived fatigue and mood of trained athletes with no clinical iron deficiency, without concurrent improvements in oxygen transport capacity or performance.

## Introduction

Iron is an essential nutrient for the optimal functioning of the human body [1]. Integral for hemoglobin synthesis, iron also plays an important role in many cellular processes; particularly, oxygen transport and storage, generation of energy through oxidative phosphorylation [1] and enzyme function affecting intracellular metabolism [2]. In addition, iron assists immune function, DNA synthesis, the functioning of neurotransmitters and the formation of myelin, and is therefore critical for normal brain function and cognitive development [3].

Iron status is influenced by both intake from dietary sources and iron loss through sweat and urine [4], gastrointestinal bleeding, menstruation and trauma. In athletes, exercise stress may exacerbate iron loss due to foot-strike hemolysis associated with running [5]. Iron deficiencies are common in athletic populations, and frequently manifest through feelings of lethargy, a lack of energy and lowered perceived ability to train and perform [4]. In clinical terms, iron deficiency is often classified as serum ferritin values below <22 µg·L^−1^, with iron deficiency anemia diagnosed when iron stores are exhausted (serum ferritin <15 µg·L^−1^), and consequently hemoglobin (Hb) levels fall below 12 g·L^−1^ [6]. However, even amongst the medical literature there remains wide debate as to the most appropriate thresholds for diagnosis and when to begin supplementation, particularly in athletes [7].

Iron supplementation, typically via the oral route, is commonly used for the treatment of iron deficiency. Recently, carbohydrate-encased intravenous (IV) compounds such as ferric carboxymaltose have proven successful in treating iron-deficiency anemia [8] and improving fatigue [9] in an outpatient setting. Krayenbuehl et al. [10] examined the effect of IV iron (III)-hydroxide sucrose on fatigue in non-anemic, premenopausal women with serum ferritin <50 µg·L^−1^ using a placebo-controlled blinded design. Reduced feelings of perceived fatigue were observed following treatment but without concomitant changes in hemoglobin concentration ([Hb]), leading the authors to speculate that the non-hematological functions of iron, namely its involvement in the formation of key enzymes involved in oxidative metabolism [11]–[13], were responsible for the observed benefits of the iron treatment.

Indeed, the chosen delivery route (intravenous as opposed to oral), and subsequent bioavailability of the supplemented iron may also be a contributing factor to relatively novel findings of the Krayenbuehl study [14]. Recently, Garvican et al. [15] compared the efficacy of 6 weeks of oral versus IV iron supplementation in distance runners with “low” (initial serum ferritin <35 µg.L^−1^ and transferrin saturation <20%, or serum ferritin <15 µg.L^−1^) or “suboptimal” (serum ferritin <65 µg.L^−1^) iron status. Whilst both forms of supplementation improved iron status, the increase in serum ferritin was greater in the IV treated group and improvements in hemoglobin mass (Hbmass), maximal aerobic power (VO_2_max) and run time to exhaustion were evident only in the IV treated athletes with initial “low” iron status. Increases in Hbmass were not observed in the “suboptimal” group suggesting that their iron stores did not limit erythropoiesis or aerobic capacity. Unfortunately, neither subjective measures nor performance *per se* were formally assessed by Garvican et al. [15], so conclusions about any other potential benefits (non-hematological or otherwise) of IV iron therapy for an athletic population could not be drawn.

At present, the optimal iron status for overall wellbeing and training ability (that is, over and above that which is required for erythropoiesis) in athletic populations is unclear. Previous investigations of the benefit of oral supplementation for endurance performance in athletes without a clinical iron deficiency, i.e. “normal” iron status, have been equivocal [4] and as a result, routine supplementation for iron-replete individuals is not encouraged. However the recent findings of Krayenbuehl et al. (2011) suggest that IV iron supplementation may afford some potential benefits unrelated to erythropoiesis or oxygen transport, due to its high bioavailability [16] compared with oral supplements [15], which may have consequences for athletic performance and thus permit the topic to be revisited. Nonetheless, the ethical and potential health implications (both long and short term) of IV iron supplementation for athletes considered “clinically normal” in terms of iron status should also be considered, particularly in regard to risk of toxicity and iron overload, and notwithstanding the difficulty in determining the appropriate IV iron dose for such individuals.

Given the efficacy of the newer formulations of iron (as opposed to oral supplementation) at improving fatigue in clinical settings [14], [17], the aim of the present study was to assess the impact of intravenous iron supplementation on perceived fatigue, mood disturbance, training ability and running performance in non-anemic athletes with clinically normal serum ferritin levels.

## Methods

### Ethics Statement

The study was approved by both the Australian Institute of Sport Human Ethics Committee and the University of Canberra Human Research Ethics Committee. All athletes provided written informed consent prior to participation.

### Study design

**Figure 1:**
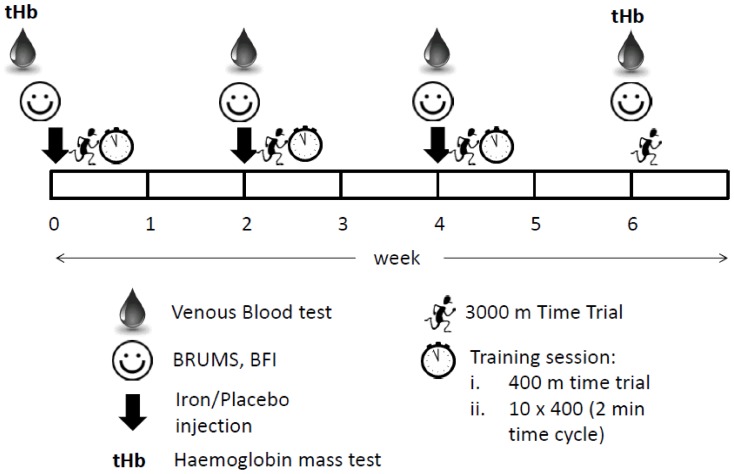
Schematic outline of the study design and testing sessions. BRUMS: Brunel mood Scale; BFI: Brief fatigue Inventory. Weekly injections performed on day 1, followed by 3000 m time trial on day 2 and monitored training session (400 m time trial/10 min recovery/10×400 m) on day 3.

Highly trained distance runners with clinically normal iron status (serum ferritin 30–100 µg.L^−1^) were randomly assigned to receive either 2 ml (100 mg) of ferric carboxymaltose (IRON) or normal saline (PLACEBO) intravenously every fortnight for four weeks. Each IV injection was closely followed by a 3000 m time trial run and a monitored training session consisting of an all-out 400 m time trial and 10×400 m training session, performed on consecutive evenings during the months of June and July in Canberra, Australia, with mean environmental temperature of 10.4°C. Iron status, mood and fatigue were examined throughout, with Hbmass assessed pre and post the intervention period (Figure 1).

### Inclusion/Exclusion criteria

Non-anemic athletes ([Hb]> 12 g.d·L^−1^) with serum ferritin between 30–100 µg·L^−1^ (determined in duplicate prior to inclusion in the study) were screened by a medical doctor to determine their suitability for participation. Athletes excluded from participation were those currently taking oral supplementation or presenting with iron deficiency anemia, anemia not related to simple iron deficiency, serum ferritin <30 µg.L^−1^ or> 100 µg.L^−1^, previously documented hypersensitivity to iron, iron overload, acute illness, renal and endocrine disorders, pregnancy, or those having completed altitude training within one month prior.

### Participants

Fourteen trained distance runners (six males, eight females) were recruited from local distance running groups (Table 1). All athletes had a minimum three-year consistent training history, and were competitive at State level or above. All athletes were familiar with the running tests employed, since similar training sessions were performed regularly within their training groups.

**Table 1:** Participant characteristics used to pair match groups at the start of the study (means ± standard deviations).

Group	Male/Female (M/F)	Age (y)	Height (cm)	Mass (kg)	Training (km·wk^−1^)	3000 m PB (s)	VO_2_max (ml·kg·min^−1^)	Hbmass (g)	Relative Hbmass (g·kg^−1^)	Serum ferritin (μg·L^−1^)
**IRON**	3M/4F	29±10	170±7	59.0±6.3	86±22	606±49	60.9±6.9	703±154	11.7±1.9	62.8±21.9
**PLACEBO**	3M/4F	28±10	172±7	62.6±8.7	70±25	606±76	59.4±6.2	760±157	12.0±1.6	62.8±26.4

IRON  =  iron supplemented group; PLACEBO  =  saline supplemented group; PB  =  personal best running time.

### Hemoglobin Mass

Hemoglobin mass was measured prior to the study, and two weeks following the final injection (week 6) using the optimized carbon monoxide (CO) rebreathing method of Schmidt and Prommer [18], whereby a 1.2 ml·kg^−1^ bolus of CO was rebreathed through a closed-system spirometer for two minutes. A CO-oximeter (OSM3, Radiometer, Copenhagen, Denmark) measured the percent carboxyhemoglobin of capillary blood at 0 and 7 minutes post-rebreathing, with five replicates of each sample performed where possible. Typical error, calculated previously from duplicate measures on a separate group of distance runners was 1.6 % (90% CL 1.3 to 2.2%) [15].

### Familiarization

One to two weeks before the study commenced, two 3000 m time trials and one 400 m time trial were performed within one week on a synthetic 400 m running track for familiarization.

### Intervention

Participants were pair-matched by an independent researcher based on their initial serum ferritin level, Hbmass, personal best time for 3000 m and aerobic fitness (VO_2_max, assessed at the start of the study as described previously [15]). Individuals were then randomly assigned to either the IRON (n = 7) or PLACEBO (n = 7) group to receive three fortnightly injections over four weeks (weeks 0, 2, 4). The IRON group received 2 ml (100 mg) of ferric carboxymaltose solution (Ferinject©, Vifor Pharma Ltd, Switzerland), whilst the corresponding PLACEBO group received 2 ml of 0.9% saline solution (Sodium chloride 0.9%).

Prior to each injection, and at least 12 h since the last training session, a venous blood sample (5 ml) was obtained via venepuncture by a trained phlebotomist to assess [Hb], hematocrit (Hct), and iron status (serum ferritin, soluble iron, transferrin and percent transferrin saturation (TSAT). Whole blood was analyzed within four hours of collection (XT-2000i, Sysmex Corporation, Japan), whilst iron status was assessed from serum (Integra 400 biochemistry analyzer, Roche Diagnostics, Switzerland). If Hct values exceeded 50%, or serum ferritin values exceeded 200 µg·L^−1^ for females, or 300 µg·L^−1^ for males, then iron supplementation would not be performed and instead replaced by saline. Each injection was performed by a medical practitioner via an indwelling venous cannula in the forearm after first flushing with 2 ml of 0.9% saline. All participants were monitored for heart rate, blood pressure, oxygen saturation and temperature by medical staff prior to administration, and every five minutes for 15 minutes afterwards in case of any adverse reactions. Throughout each injection, the participant's arm was shielded using a screen with the injection solution prepared out of sight. In addition to all athletes, all research staff associated with the testing and training sessions were blinded to the treatment groups and were unable to access blood results, nor preside over the injections. Thus, only the medical staff administering the injections had access to grouping allocation information.

### Fatigue and Mood Questionnaires

Participants were assessed fortnightly for six weeks for their perceptions of fatigue and mood disturbance using the Brief Fatigue Inventory (BFI) [19] and Brunel Mood Scale (BRUMS) [20], [21] (Figure 1). The BFI consists of nine standardized questions that participants answered on a scale of 1 “No fatigue” to 10 “As bad as you can imagine”, as previously detailed [10]. The resulting scores from each question were averaged to produce a Total Fatigue Score.

Mood was assessed using the Brunel Mood Scale (BRUMS) [20], [21], a 24-item questionnaire for assessment of mood descriptors including Tension, Depression, Anger, Fatigue, Vigor and Confusion. Each item was anchored by a 5-point Likert scale from 0 “Not at all” to 4 “Extremely” according to how participants feel “right now”. Raw scores for each question and the related mood descriptor were converted to standard T-scores using normative data [22] to produce a final score, with Total Mood Disturbance then calculated by adding each of the scores for the mood descriptors and subtracting the score for Vigor [23].

### Performance Testing Set: 3000 m Time Trial

The day following each injection (week 0, 2, and 4), and approximately two weeks after the completion of the supplementation period (week 6 follow-up), participants completed a maximal effort 3000 m time trial individually on a synthetic outdoor 400 m (7.5 laps) running track (Figure 1) to assess their maximal running performance capability. The 3000 m distance was chosen since it is run at speeds associated with VO_2_max, and has been shown previously to be a reliable indicator of running performance in trained runners [24]. Athletes were familiar with the protocol and were blinded to pace and time during the effort. Total time was recorded via a manual stopwatch (S056-4000, Seiko, Japan). BLa was measured one minute post-trial, as well as RPE using the 6-20 Borg Scale [25].

### Monitored Training Set: 10×400 m

In order to assess the athletes' ability to recover from the time trial, and their subsequent readiness to train, a monitored training session was completed 22–24 hours after the 3000 m time trial at week 0, 2 and 4. The timeframe between the time trial and monitored training session was ″24 h shorter than typically experienced by the athletes, but is reflective of competition schedules at major championship meets where athletes may be required to complete heats and finals on consecutive days. After a standardized self-selected warm-up, participants completed an all-out 400 m time trial individually. BLa and RPE were recorded three minutes post-trial. After 10 minutes of active recovery, athletes undertook as a group a training session consisting of 10×400 m, on a two minute time cycle (Figure 1), with individual lap times recorded via manual stopwatch. Mean lap time was calculated as the average of the 10 lap times. BLa was measured one minute after completion of the final 400 m lap. After 10 min, session RPE was obtained using the 1–10 Borg Scale for Session RPE (sRPE) [26].

### Training and Lifestyle

Participants were asked to record their training daily using an electronic training diary for the duration of the study period. In addition to recording total distance and training time, they were asked to rate their feelings related to “*How ready do you feel to train today? How easy was it to get out of bed this morning? How energized do you feel today? How much did you give in your training today? How sore are you feeling?*” on a scale of 1–5. Athletes were also instructed not to change their normal dietary practices, and to refrain from taking any oral iron supplements.

### Statistics

Potential differences between groups at baseline were assessed using a Student's t-test, with significance set to p≤0.05. Otherwise, magnitude-based inferences were used in order to define the practical significance of the results using the unequal variances t-statistic and Cohen's Effect Sizes (ES) [27], [28]. The differences and associated 90% confidence limits (CL) for changes in hematological, physiological and performance characteristics were examined *within* each group over the duration of the study, as well as *between* the groups for the differences in their respective changes from baseline measures. Prior to analysis, raw data were log transformed to reduce non-uniformity of errors. The magnitude of an effect and the associated P-value was presented as percent change and standardized mean differences (ES); calculated as the difference in mean divided by the between-subject standard deviation (SD); where a small effect is> 0.2, moderate> 0.6 and large> 1.2 [27]. The reference threshold for the smallest worthwhile change was set to 0.2, which is Cohen's smallest standardized effect calculated from the between-subject SD at baseline. Effects were deemed *unclear* if the confidence interval overlapped the thresholds for the smallest positive and negative effects. Data are reported as mean ± SD unless otherwise stated.

The overall change in the subjective parameters of the training diaries was assessed with within-individual linear regressions (change/day, with 90% CL) as described by others [29]. Trends were deemed substantial if the 90% CL did not overlap zero. Differences of the slopes between the IRON and PLACEBO groups were also assessed using magnitude based inferences as described above [27].

## Results

### Study Population

**Figure 2:**
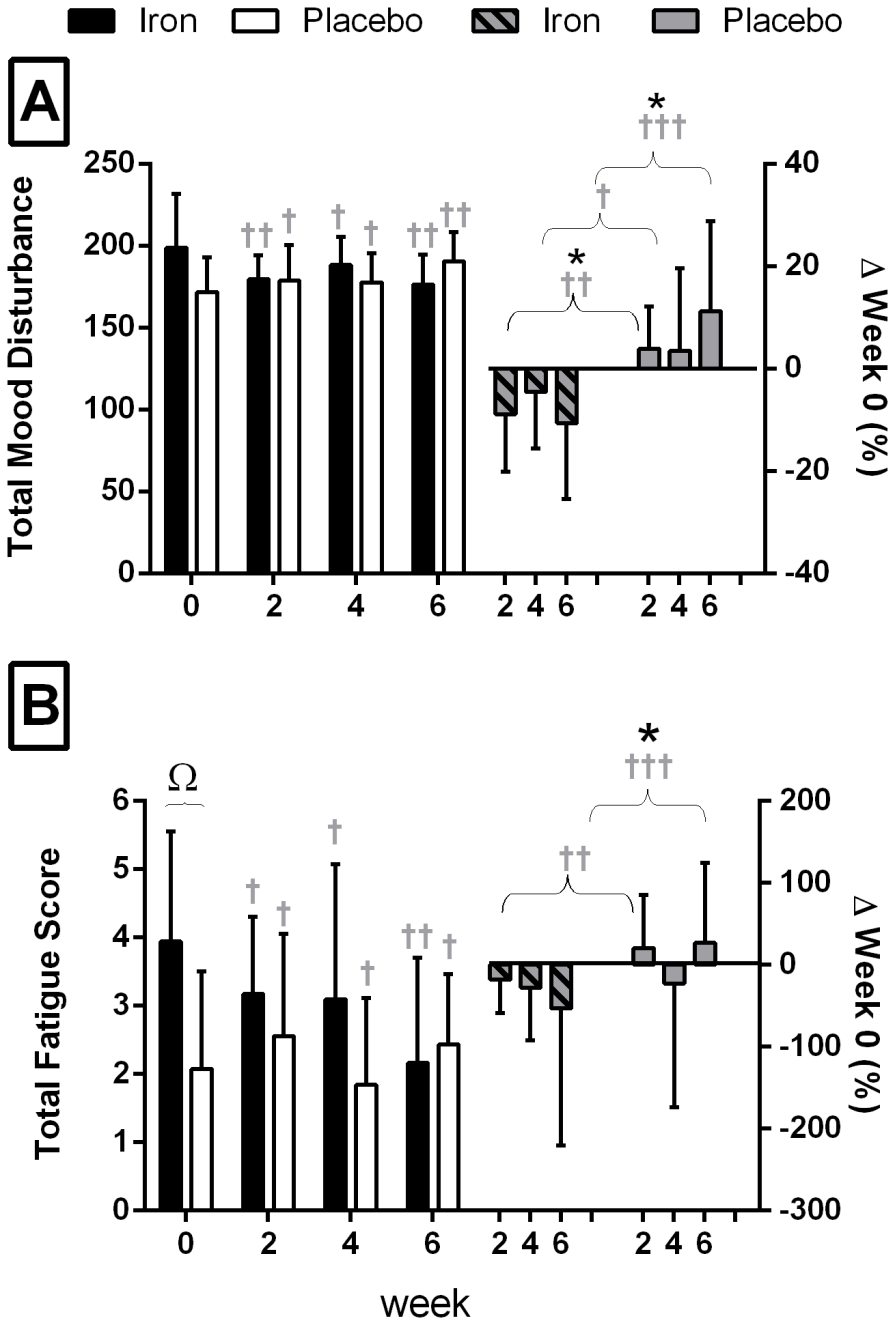
Changes in mood and fatigue scores during the study. **A)** Total Mood Disturbance from the Brunel Mood Scale and **B)** Total Fatigue Score from the Brief Fatigue Inventory. Data are presented in raw form on the Left Y-axis and as percent change from week 0 on the Right Y-axis (mean ± SD). Within a group change from Week 0: † small effect (Effect size> 0.2), †† moderate effect (Effect size>0.6); between group percent change from week 0 at matched time; * p≤0.05; † small effect (Effect size> 0.2), †† moderate effect (Effect size> 0.6), ††† Large effect (Effect size> 1.2); Ω denotes difference between groups at week 0.

There were no significant differences between the IRON and PLACEBO groups at the start of the study in terms of baseline characteristics (Table 1), hematological parameters (Table 2), or mood scores associated with the BRUMS (Figure 2A). However at baseline, IRON had a significantly higher Total Fatigue Score than PLACEBO on the BFI (Figure 2B, p = 0.04).

**Table 2:** Iron parameters, Hbmass,[Hb] and fatigue scores of IRON and PLACEBO groups pre-supplementation and at 2, 4 and 6 weeks post-supplementation.

	IRON				PLACEBO			
	Week 0	Week 2	Week 4	Week 6	Week 0	Week 2	Week 4	Week 6
**Serum ferritin (μg·L^−1^)**	62.8±21.9	104.1±31.7 *†	128.1±46.6 *†	127.0±66.3 *†	62.8±26.4	65.7±30.1	65.8±26.4	57.3±17.7
**Hbmass (g)**	703±154	-	-	709±148	759±157	-	-	758±170
**Relative Hbmass (g·kg^−1^)**	11.7±1.9	-	-	11.8±1.6	12.0±1.6	-	-	12.1±1.6
**[Hb] (g·dL^−1^)**	14.6±1.6	14.2±1.3	14.3±1.5	14.4±1.1	14.6±1.0	14.3±1.2	14.4±1.0	14.3±1.0
**Hct**	42.8±3.8	41.7±2.9	41.9±3.8	42.0±2.4	42.5±2.8	42.5±2.7	42.2±1.8	41.6±2.2
**Iron (μmol·L^−1^)**	19.8±4.3	18.2±7.0	16.1±3.8	17.2±6.6	17.5±3.5	15.1±4.2	18.5±4.6	16.6±7.5
**Transferrin (g·L^−1^)**	3.3±0.4	2.8±0.4 *	2.8±0.6 *	2.7±0.4 *	3.1±0.3	2.8±0.2	2.9±0.2	2.8±0.3
**TSAT (%)**	23.0±4.6	25.0±9.1	23.1±7.0	23.6±8.3	21.7±5.7	20.6±6.1	24.3±6.7	22.0±9.3

Mean ± SD; * denotes significant difference (p≤0.05) from week 0 values within a group.

† significant difference (p≤0.05) between groups at matched time point.

Hbmass: haemoglobin mass, [Hb]: haemoglobin concentration, Hct: Haematocrit, TSAT: percent transferrin saturation.

### Supplementation, Hematology and Iron profile

Each scheduled injection was administered without incident. Serum ferritin values did not change significantly in PLACEBO but increased two-fold from baseline in the IRON group at week 4 (Week 0: 62.8±21.9, Week 4: 128.1±46.6 µg·L^−1^; p = 0.002) and remained elevated two weeks after the final injection (127.0±66.3 µg·L^−1^; p = 0.01; Table 2). There were no statistically significant changes in Hbmass, [Hb] or Hct in either group over the course of the investigation (Table 2).

### Fatigue Scores

#### Brunel Mood Scale

Iron supplementation had a moderate (but not significant) effect on Total Mood Disturbance (Figure 2A), with values within the IRON group tending to be lower than baseline at week 2 (ES –0.64, p = 0.06) and 6 (ES –0.77, p = 0.08). Between the groups, Total Mood Disturbance significantly improved in IRON compared with PLACEBO (Figure 2A), at both week 2 (% change compared with PLACEBO; 90% CL, ES, p value: −12.3%; −19.8 to −4.1%, −0.95, p = 0.02) and week 6 (−19.6%; −30.4 to −7.1%, −1.58, p = 0.02).

#### Brief Fatigue Inventory

Within the IRON group, supplementation resulted in a trend to decrease the Total Fatigue Score at week 6 (ES -1.35, p = 0.09), but the latter large effect size failed to attain statistical significance (Figure 2B). Between the groups, Total Fatigue Score in the IRON group significantly improved at week 6 compared to PLACEBO (−63.0%; −83.7 to −16.1%, −1.54, p = 0.05).

### Running performance

#### 3000 m Time Trial

**Figure 3:**
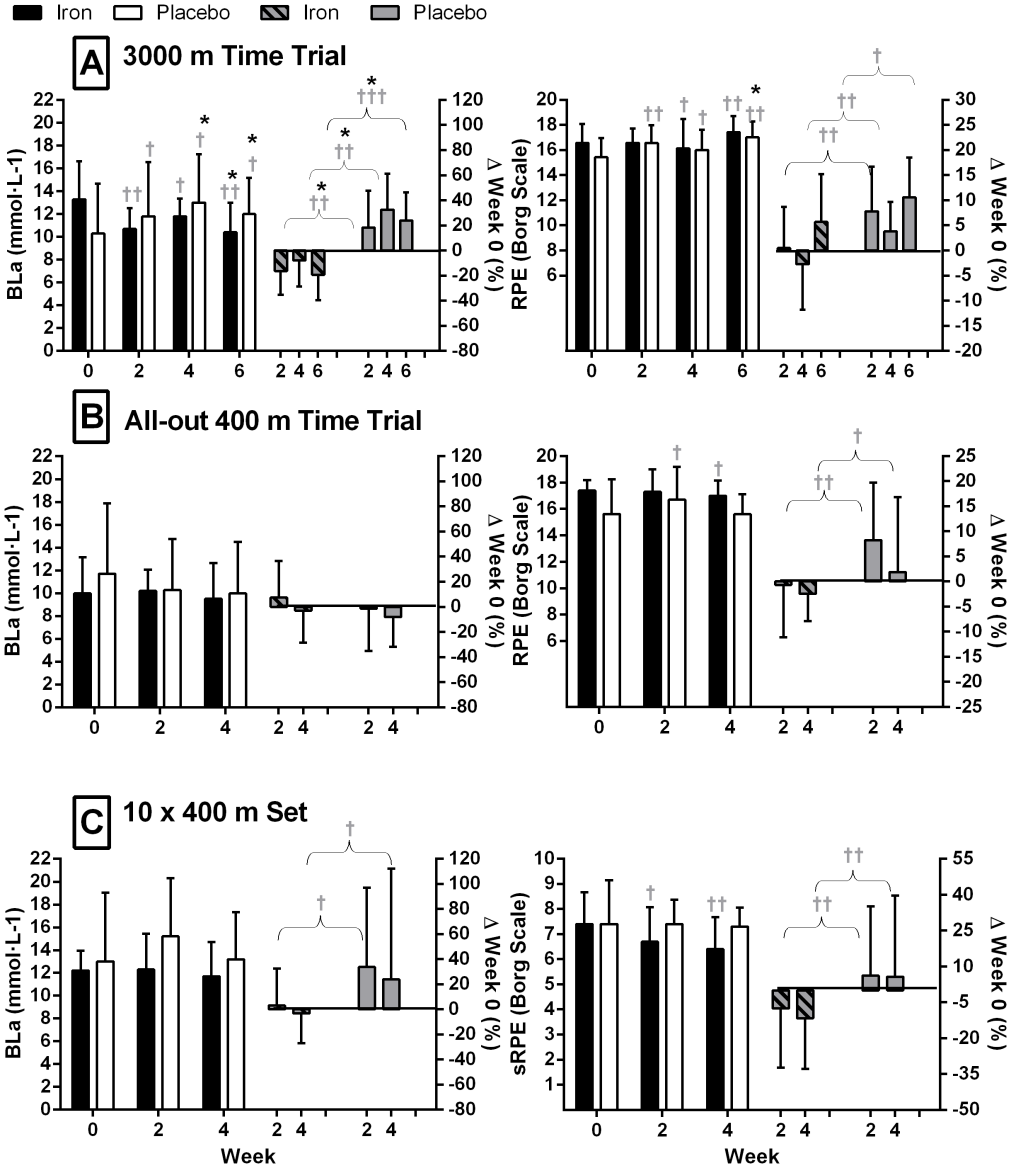
Blood lactate concentration (Bla) and Rating of Perceived Exertion (RPE) from the run trials over the course of the study. **A)** 3000 m time trial; **B)** All-out 400 m time trial; **C)** 10×400 m session. Data are presented in raw form on the Left Y-axis and as % change from week 0 on the Right Y-axis (mean ± SD). Within a group change from Week 0: * p≤0.05; † small effect (Effect size> 0.2), †† moderate effect (Effect size> 0.6); between group percent change from PRE at matched time; † small effect (Effect size> 0.2), †† moderate effect (Effect size> 0.6).

Mean 3000 m run time did not change significantly within IRON during the study but PLACEBO were slower than week 0 at week 4 (p = 0.04). Therefore, at week 4 there was a small effect on 3000 m time between the groups, with IRON faster than PLACEBO (−2.1 %; −3.8 to 0.3 %, 0.22; p = 0.05); Table 3). Compared with PLACEBO, the BLa of IRON was reduced at week 2 (−29.5%; −43.7 to −11.7%, −1.00, p = 0.02), week 4 (−30.8%; −44.0 to −14.5%, −1.06, p = 0.01) and week 6 (−35.8%; −48.4 to −20.1%, −1.27, p = 0.003; Figure 3A). Between the groups, iron supplementation had no significant effect on RPE.

**Table 3:** Performance test times (mean ± SD) and mean difference (with 90% CL) between groups (IRON vs. PLACEBO) for the respective percent change from week 0.

Performance test	Group	Week 0	Week 2	Week 4	Week 6 (Follow-up)	Week 2 Difference in change between groups (%)	Week 4 Difference in change between groups (%)	Week 6 Difference in change between groups (%)
**3000 m time trial (s)**	Iron	625.6±55.5	627.1±55.3	625.4±52.7	631.3±49.7	−1.2 (−2.8 to 0.3)	−2.1 (−3.8 to −0.3) †	−0.9 (−3.7 to 1.9)
	Placebo	624.8±47.2	634.9±55.2	639.1±59.7 * Ω	638.0±63.2 †			
**All-out 400 m time trial (s)**	Iron	67.5±6.2	68.0±6.6	68.8±6.1	-	0.6 (−2.9 to 4.2)	−0.1 (−2.7 to 2.6)	-
	Placebo	66.9±9.8	66.7±7.5	68.2±9.4	-			
**10×400 m Average time (s)**	Iron	78.0±6.6	77.2±6.3 *	77.1±5.7	-	−0.7 (−4.3 to 2.0)	−1.8 (−5.5 to 2.1) †	-
	Placebo	78.4±7.3	78.2±6.2	78.9±7.6	-			

Within group change from Week 0: * p≤0.05, Ω small effect (Effect size> 0.2).

Between groups change from Week 0: † small effect (Effect size> 0.2).

#### All-out 400 m Time Trial

All-out 400 m time did not change significantly within or between either group over the course of the study (Table 3). Differences between the groups for changes in BLa and RPE were not significant (Figure 3B).

#### 10×400 m Training Session

The average time for the 10×400 m session of the IRON group was faster at week 2 (−0.8%; −1.2 to −0.5%, −0.09, p = 0.004) compared with week 0. There were no significant differences within PLACEBO over time (Table 3), nor were there any significant differences between groups. Between the groups, iron supplementation had no significant effect on RPE or BLa (Figure 3C).

### Training Diaries

**Figure 4:**
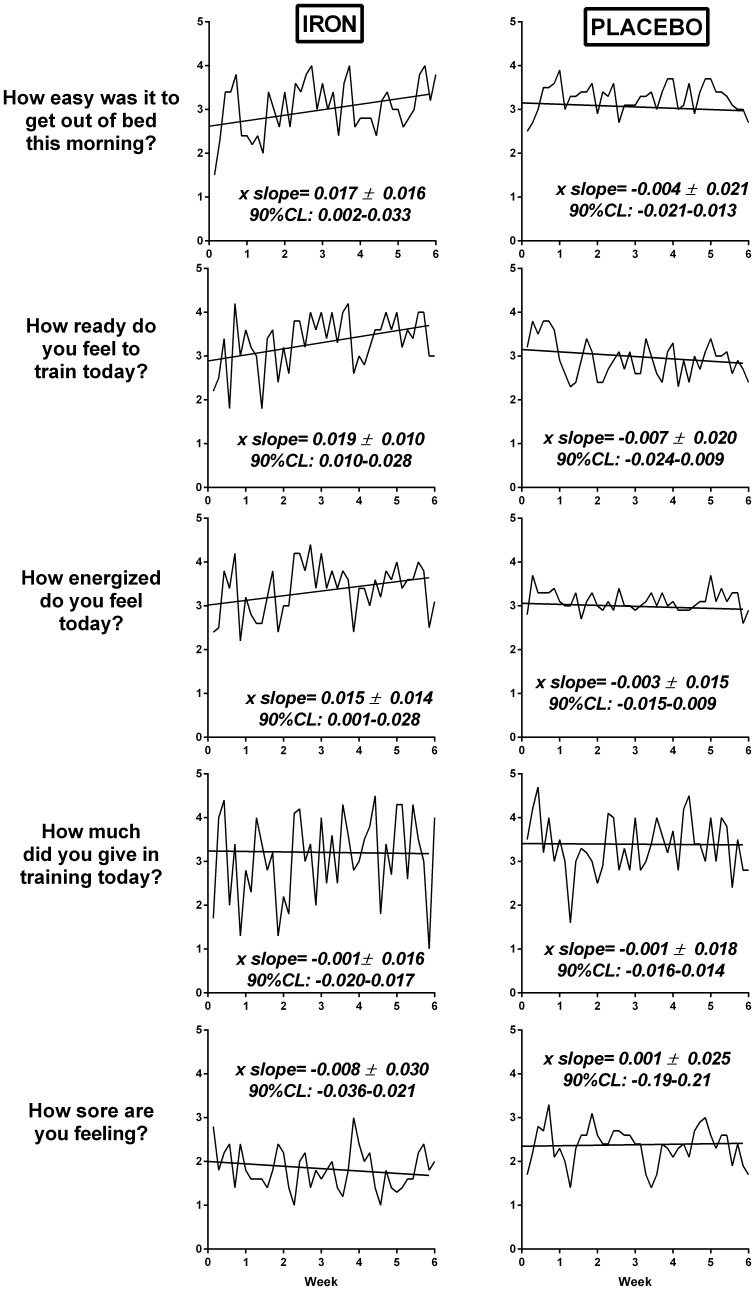
Mean perceptual daily training diary ratings for IRON and PLACEBO during the study (rated 1 to 5). X slope (90%CL): derived as the mean of individual regression analysis for each subject.

Day-to-day mean training diary scores improved in the following areas within the IRON group only: “How easy was it to get out of bed this morning?”, “How ready do you feel to train today?”, and “How energized do you feel today?” (Figure 4). The observed positive effects for these questions were greater in IRON compared with PLACEBO (Effect size; 90CL: “How easy was it to get out of bed this morning?”0.88; 0.04 to 1.71; “How ready do you feel to train today?”1.12; 0.38 to 1.86, and “How energized do you feel today?” 1.04; 0.11 to 1.97).

## Discussion

The main finding of the present study was that, compared with PLACEBO, three fortnightly IV injections of ferric carboxymaltose over 4 weeks improved self-reported perceptions of fatigue and reduced mood disturbance in trained distance runners with “clinically normal”serum ferritin levels between 30 and 100 µg·L^−1^, without concomitant changes in Hbmass or [Hb]. Supplementation had no acute effects on 3000 m running performance; however iron supplemented athletes decreased their 10×400 m training set time at week 2, and recorded improvements in their perceptions about their preparedness to train, which collectively may indicate a potential training benefit of IV iron for the treatment group.

The most widely accepted clinical threshold for iron deficiency is 22 µg·L^−1^ [30], where storage iron is exhausted but transport and functional compounds are maintained. When ferritin stores fall below 22 µg·L^−1^, functional compounds become compromised, including but not limited to hemoglobin, myoglobin and cytochromes [30]. However, the “optimal” level for serum ferritin is still unknown in terms of athletic performance and an athlete's ability to train and recover between sessions over weeks or months. Uncertainty is due largely to discrepancies in the literature regarding the effect of iron supplementation, particularly in athletes with ‘normal’ iron status [2], and hampered by a wide variety of supplementation methods and protocols [7]. In the present study, we specifically recruited subjects with clinically normal ferritin levels (30–100 µg·L^−1^), whose Hbmass was therefore unlikely to be compromised [15], in order to investigate possible non-hematological effects of IV iron supplementation; that is, those unrelated to erythropoiesis or oxygen transport, such as subjective wellbeing and training ability.

Intravenous ferric carboxymaltose supplementation increased serum ferritin throughout the supplementation period. Further, serum ferritin remained elevated by approximately double the pre-treatment levels two weeks following the final injection. However, Hbmass and [Hb] did not change, confirming previous research [15] but also indicating that the observed reductions in fatigue and mood disturbance in the iron treatment group, compared to placebo, were unlikely to be related to oxygen-carrying capacity.

When storage iron is> 35 µg·L^−1^, erythropoiesis and thereby oxygen transport, are unlikely to be affected [15]. However, it is possible that if the amount of iron available for the formation of enzymes and compounds associated with muscle metabolism is insufficient, then endurance performance may also become impaired [31]. Since it would appear that the supplemental iron provided in the present study was not required for the formation of Hb, it is possible that the available iron may have been shuttled towards oxidative enzymes for intracellular metabolism and electron transport [1]. However, in the present study, 3000 m running time was not acutely improved following each injection; indicating neither a direct performance advantage following IV iron administration nor a placebo effect of an injection. Further, although subtle improvements in the 10×400 m training set were observed in the IRON group at week 2, overall our results do not provide compelling evidence for any performance advantage arising from increasing ferritin stores. Of note, however, is the observation that 3000 m performance was worse in the PLACEBO group at week 4, and as a result the IRON group performed significantly better. These findings provide tentative evidence that the IRON group performed more consistently throughout the study, and may be related to improved perceptions of fatigue and well-being as opposed to effects at a metabolic level.

The presence of blood lactate, a gross indirect measure of anaerobic metabolism, reflects the contribution of each of the body's energy systems with which an exercise effort is undertaken. A decrease in BLa within the IRON group was observed following the week 2 and week 6, 3000 m time trials, which is similar to previous findings [32]–[34], whilst PLACEBO recorded higher BLa for comparable run times. Hinton et al. [35] report that as a result of iron deficits, the oxidative capacity of the muscle is reduced and therefore the workload at which the ventilatory threshold and blood lactate accumulation occurs is lower [35]. Some studies have reported no change in BLa following supplementation [36], [37], but discrepancies in time course of treatment and the chosen modality (most have involved oral supplementation), dosage and study design may have influenced these results. The iron recipients in the present study may thus have improved aspects of their lactate metabolism [1], [34] as a result of treatment effects at a metabolic level, although further investigation is required to confirm this notion.

Iron deficiency without anemia can result in a greater energy expenditure during a simulated time trial [38] as well as lower exercise tolerance and a more prolonged recovery from exercise [39]. The IRON group decreased their average 10×400 m time shortly after the second injection, even though their 3000 m and all-out 400 m times did not improve. These results partially support the notion of resilience to an exercise bout and weakly suggest the iron supplemented group may have displayed a greater ability to complete consecutive training sessions whilst carrying potential fatigue from a prior effort. It is possible that improved iron stores may result in greater coping ability and enhanced recovery before the next session, which may in turn facilitate augmented wellbeing and ability to train more effectively, although further research is required.

There is evidence that iron insufficiencies impact mental function and emotional health as a result of reduced neurotransmitter activity in the brain and central nervous system [1]. Iron therapy can result in improved cognitive function [40] and has been associated with improvements in memory, energy and mood [41], as well as improvement in subjective feelings of physical fitness and performance [42], [43]. This is supported by data in the present study where the recipients of IV iron reported improvement in total fatigue and total mood disturbance, in addition to an improvement in their training diary scores relating to readiness to train, energy levels and ease of getting out of bed. By contrast, some studies have reported that iron supplementation has no significant effect on deficiency symptoms, mood state [44] or quality of life [45]. The discrepancy may be due to the oral treatment modality examined by earlier researchers, which may not deliver enough bioavailable iron for substantial cognitive improvements.

### Limitations

Despite careful pair matching of subjects based on initial ferritin, Hbmass and performance characteristics, the PLACEBO group reported lower fatigue scores on the one of the two questionnaire scales (BFI) at the start of the study. The BFI is a tool typically used to assess cancer-related fatigue but has been used previously in a non-athletic population to assess iron related fatigue [14]; whereas the BRUMS was developed for assessment of mood (including fatigue) in performance environments. The difference in baseline scores using the BFI highlights that the presence of fatigue symptoms is not solely related to initial ferritin level. Nonetheless, when the difference in change scores of both scales (BRUMS and BFI) was analyzed, a superiority of the IRON group over the PLACEBO group was observed, indicating a possible role of supplemental iron in the reduction of perceived fatigue and an improvement in overall mood. It should also be noted that whilst improvements were observed in the self-reported training diary scores of the IRON group, this method of reporting is only typically used in an applied sport model as opposed to a clinical setting.

No clear acute or chronic benefits of iron supplementation on 3000 m running performance were observed although consistently similar performances were evident throughout which was not the case in the PLACEBO group. Measuring a true maximal effort of an individual as well as obtaining optimal performance on a particular day, remain inherently difficult [46]. The very nature of simulated performance testing means that is nearly impossible to mimic real-life competition through a time trial or simulated laboratory test, which may explain some early research reporting no improvement in performance post-supplementation even in iron-deficient participants [36], [37], [47]. A further limitation to consider is that our total monitoring period was possibly too short, particularly since we did not follow the 10×400 m training set at two weeks post injection.

### Implications

This study demonstrated improvements in fatigue and mood disturbance in the IRON group during a 6 week training period. Whether or not such improvements in fatigue and mood (potentially arising from elevated ferritin levels) may lead to better training quality and subsequently improved performance over a longer period of time cannot be determined from this study. However, the long term or repeated use of iron supplementation in athletes who are not iron deficient should not be advocated. In the short-term, the efficacy of IV Iron supplementation for athletes with moderate ferritin levels also requires careful consideration, because a performance improvement remains unclear. For this reason the data in this study do not provide strong evidence for IV iron supplementation in athletes with clinically normal ferritin levels, especially when taking into account the ethical and potential health implications of sustained iron supplementation for iron replete individuals. Specifically, the potential risk of toxicity and iron overload following IV administration must be acknowledged, particularly since the primary route for regulating whole body iron stores is bypassed using this method, allowing ferritin levels to be elevated rapidly. Therefore, IV injections should not be administered without first assessing existing iron stores, and careful consideration of the total dose to be given, and its likely impact on whole body iron stores, is required. IV iron should only be administered under medical supervision, with patients closely monitored for adverse reactions. Follow-up tests should also be conducted to ensure ferritin levels do not exceed healthy limits. Lastly, researchers need to be acutely up to date with any changes to the World Anti-Doping Agency code when planning future research with elite athletes [48].

## Conclusion

Four weeks of IV ferric carboxymaltose supplementation doubled serum ferritin, and decreased feelings of fatigue and mood disturbance in non-anemic athletes with initial ferritin levels in the clinically normal range of 30–100 µg·L^−1^ without associated changes in Hbmass. Running performance was not acutely affected by iron supplementation, but there was some evidence from self-reported training diaries that participants felt more able to train. Together, these results indicate a potential psychobiological influence of iron supplementation on feelings of mood and fatigue in athletic populations with clinically normal serum ferritin.
